# Model-inferred mechanisms of liver function from magnetic resonance imaging data: Validation and variation across a clinically relevant cohort

**DOI:** 10.1371/journal.pcbi.1007157

**Published:** 2019-06-25

**Authors:** Mikael F. Forsgren, Markus Karlsson, Olof Dahlqvist Leinhard, Nils Dahlström, Bengt Norén, Thobias Romu, Simone Ignatova, Mattias Ekstedt, Stergios Kechagias, Peter Lundberg, Gunnar Cedersund

**Affiliations:** 1 Wolfram MathCore AB and Department of Medical and Health Sciences, Linköping University, Linköping, Sweden; 2 Center for Medical Image Science and Visualization (CMIV), Linköping University, Linköping, Sweden; 3 Department of Medical and Health Sciences, Linköping University, Linköping, Sweden; 4 Department of Radiology, Department of Medical and Health Sciences, Linköping University, Linköping, Sweden; 5 Department of Biomedical Engineering, Linköping University, Linköping, Sweden; 6 Department of Clinical Pathology and Clinical Genetics, Department of Clinical and Experimental Medicine, Linköping University, Linköping, Sweden; 7 Department of Gastroenterology and Hepatology, Department of Medical and Health Sciences, Linköping University, Linköping, Sweden; 8 Department of Radiation Physics, Department of Medical and Health Sciences, Linköping University, Linköping, Sweden; 9 Department of Clinical and Experimental Medicine, Linköping University, Linköping, Sweden; University at Buffalo - The State University of New York, UNITED STATES

## Abstract

Estimation of liver function is important to monitor progression of chronic liver disease (CLD). A promising method is magnetic resonance imaging (MRI) combined with gadoxetate, a liver-specific contrast agent. For this method, we have previously developed a model for an average healthy human. Herein, we extended this model, by combining it with a patient-specific non-linear mixed-effects modeling framework. We validated the model by recruiting 100 patients with CLD of varying severity and etiologies. The model explained all MRI data and adequately predicted both timepoints saved for validation and gadoxetate concentrations in both plasma and biopsies. The validated model provides a new and deeper look into how the mechanisms of liver function vary across a wide variety of liver diseases. The basic mechanisms remain the same, but increasing fibrosis reduces uptake and increases excretion of gadoxetate. These mechanisms are shared across many liver functions and can now be estimated from standard clinical images.

## Author summary

Being able to accurately and reliably estimate liver function is important when monitoring the progression of patients with liver disease, as well as when identifying drug-induced liver injury during drug development. A promising method for quantifying liver function is to use magnetic resonance imaging combined with gadoxetate. Gadoxetate is a liver-specific contrast agent, which is taken up by the hepatocytes and excreted into the bile. We have previously developed a mechanistic model for gadoxetate dynamics using averaged data from healthy volunteers. In this work, we extended our model with a non-linear mixed-effects modeling framework to give patient-specific estimates of the gadoxetate transport-rates. We validated the model by recruiting 100 patients with liver disease, covering a range of severity and etiologies. All patients underwent an MRI-examination and provided both blood and liver biopsies. Our validated model provides a new and deeper look into how the mechanisms of liver function varies across a wide variety of liver diseases. The basic mechanisms remain the same, but increasing fibrosis reduces uptake and increases excretion of gadoxetate.

## Introduction

Measurements of liver function are important to determine the optimal therapeutic strategy in cases of severe chronic liver disease (CLD), and for prevention of post-treatment hepatic failure [1]. Estimating liver function is also important when planning surgical treatment, because postoperative hepatic function insufficiency is associated with both morbidity and mortality [2]. Sensitive biomarkers for liver function would also be useful for the management and early identification of drug-induced liver injury (DILI), which is a leading cause of acute liver failure [3] and also of drugs being withdrawn from the market [4].

Different options for estimation of liver function are used clinically today, but they all have some shortcomings. For instance, the primary clinical screening tool for liver injury in clinical trials, serum alanine aminotransferase (ALT), neither indicates the severity of liver injury nor estimates liver function [5]. In addition, ALT (and other transaminases) only indicates injury at a late stage when substantial tissue damage has already occurred [6]. Alternative methods for measuring liver function include Indocyanine-Green 15 retention rate (ICGR15) [7] and Tc-99m galactosyl human serum albumin (GSA) [8], which both measure the liver´s capacity to clear substances from the blood, and the galactose breath test [9], which measures the liver’s metabolic capacity. These are all examples of global indicators that provide indirect measurements of liver function. Furthermore, GSA involves the injection of a radioactive isotope, which from a practical point of view is cumbersome and suffers from limited spatial and temporal resolution, and importantly is not widely available. In summary, biomarkers that are sensitive and respond early to changes in liver function would be beneficial both in a clinical setting as well as in the pharmaceutical industry and regulatory agencies [10, 11]. Because of the low quality of available measures of liver function, little is known about the more detailed mechanisms of liver function, and about how these mechanisms change at different stages of CLD.

One of the most promising state-of-the-art methods for assessing clearance-based liver function is to use magnetic resonance imaging (MRI) in combination with the liver-specific contrast agent gadoxetate (Bayer Schering Pharma, Berlin, Germany). This method has the potential to allow investigation of liver function at a regional level without the need for any ionizing radiation. As a liver-specific contrast agent, gadoxetate is actively accumulated within hepatocytes [12] and is commonly used for characterizing lesions. The gadoxetate uptake is mainly associated with the function of the organic anion-transporting polypeptide 1 (OATP1) family of transporters [13]. The subsequent excretion into the bile occurs via the multidrug resistance-associated protein 2 (MRP2) transporter [14]. These transporter proteins have important functions, such as mediating the clearance of bilirubin, toxins, drugs, and other organic solutes [15]. For these reasons, gadoxetate MRI has the potential to facilitate study of the aspect of liver function that has to do with these uptake and excretion processes.

There are a number of previous studies which indicate the high potential of gadoxetate MRI as a biomarker for liver function. Early studies established a correlation between gadoxetate MRI and common clinical markers for liver function [16]. A more recent study demonstrates the ability of gadoxetate MRI to predict liver failure after surgery [17]. Furthermore, a recent prospective follow-up study on patients with primary sclerosing cholangitis showed that quantitative gadoxetate MRI could predict solid clinical endpoints, such as liver transplantation, cholangiocarcinoma, and liver related death [18]. The approach used in that study separated the population into two clear groups, one with >90% survival and one with <60% survival. Finally, in rats, similar analyses have shown promising results of using gadoxetate MRI as a biomarker for DILI [19, 20].

All the above clinical studies have used a simple analysis, called relative enhancement, which simply compares signal intensity before and after gadoxetate injection; therefore, the studies could not elucidate the detailed mechanisms of liver function. More advanced approaches can make use of the information in an entire time series of images and use this to extract different gadoxetate transport rates. Such methods require the use of mathematical models. One common class of such models is the perfusion-based model [21, 22]. These models require images with a very high temporal resolution, which limits the spatial resolution, meaning that the images cannot be used for conventional radiological reading. An alternative to perfusion-based models is models based on simulation and optimization of ordinary differential equations. These models do not need such high temporal resolution, but can utilize the high-spatial low-temporal resolution images used in clinical MRI protocols today. One such model, describing how gadoxetate is distributed in the whole body, as well as taken up and excreted by the liver, was described by Forsgren and colleagues [23].

**Fig 1:**
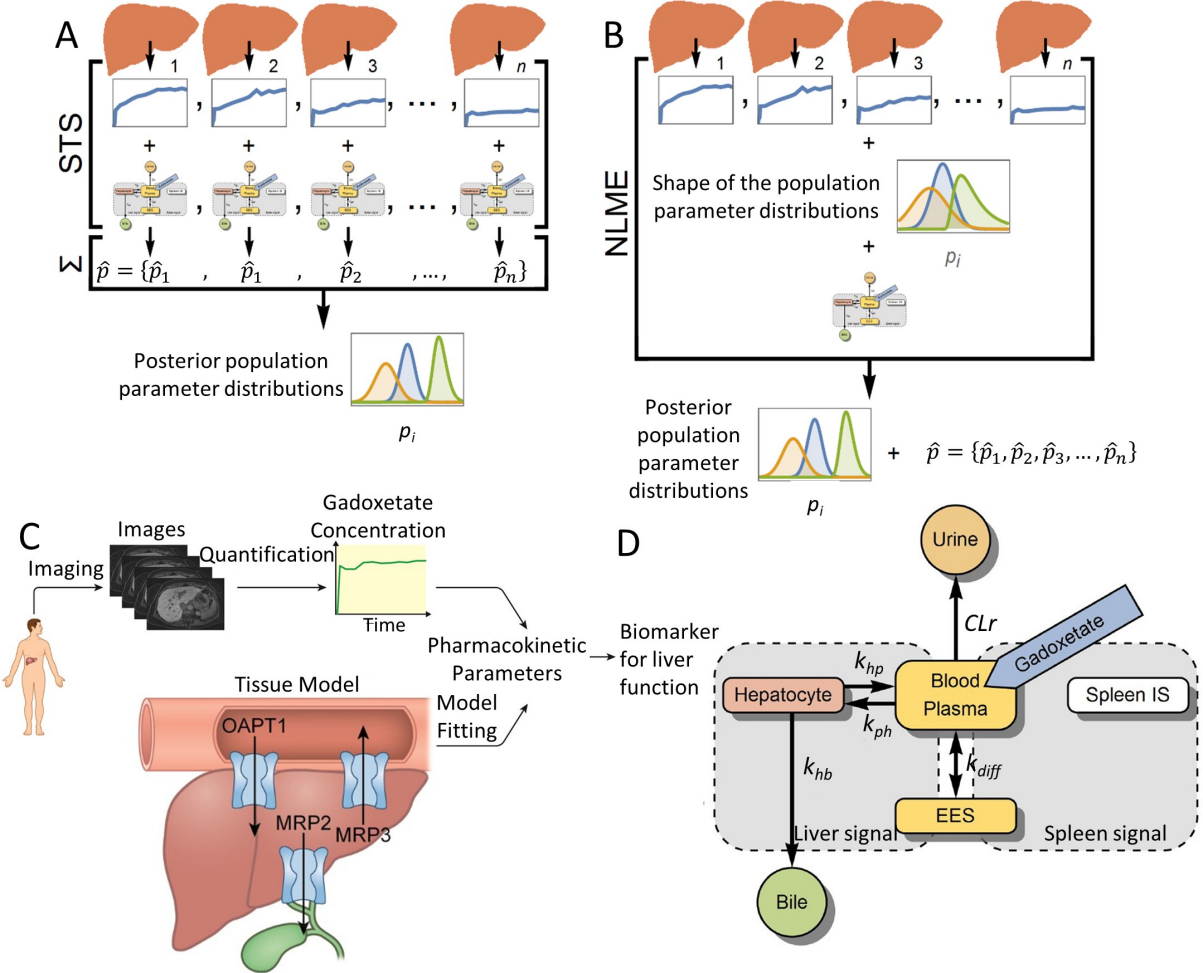
NLME, the mechanistic model framework, and model. (**A**) The standard-two-stage (STS) method. The model is parametrized for each data set separately, and then the parameter values are combined to derive population parameter distributions. (**B**) The ‘non-linear mixed effect’ (NLME) method. The shapes of the population parameter distributions are first postulated, then distributions are parametrized to all datasets, and finally each patient is parametrized following the population parameter distributions with a joint-likelihood function. This allows NLME to use the global information obtained from an entire cohort, which is utilized to improve model parametrization for each individual subject. (**C**) The framework consists of gadoxetate-enhanced images, which are processed to obtain gadoxetate concentrations in the liver. A mechanistic systems pharmacology model, describing how gadoxetate is taken up and excreted, is fitted to the data using NLME parameterization to obtain reliable pharmacokinetic parameters, which can be used as biomarkers for liver function. (**D)** Schematic diagram of the mechanistic model for quantification of liver transporter function. Rounded rectangles represent compartments in the model, with arrows indicating gadoxetate fluxes between blood plasma and extracellular extravascular space (EES; k_diff_), elimination via the kidneys to urine (CLr), uptake into hepatocytes (k_ph_), back-flux from hepatocytes into blood plasma (k_hp_), and excretion from hepatocytes into bile (k_hb_). Gadoxetate injection into the blood-plasma compartment is indicated in blue. Gray areas show the signal part of the model in which compartmental gadoxetate concentrations are combined to predict the information in the gadoxetate-enhanced MRI time series.

While the Forsgren model can arguably be viewed as the most realistic gadoxetate uptake model, it has several shortcomings. The model is the most realistic in the sense that it is the only compartment model to describe the dynamics in liver, blood, spleen, and extracellular extravascular space. Furthermore, the model has been validated in healthy humans with gadoxetate doses up to 20 times higher than the clinical dose used for model training. On the other hand, the model has not been personalized. One effective approach used for such personalization is non-linear mixed-effects modeling (NLME; Fig 1A and 1B). NLME is effective because it can deal with low-informative data [24], which could allow for fewer images, shorter clinical examinations, and more reliable parameters. However, NLME has not been applied to any gadoxetate uptake model. Another shortcoming with the Forsgren model is that it has not been tested in patients with liver diseases. Therefore, it is not known how the mechanisms in the model vary across different stages and etiologies of CLD. Furthermore, the model has not been validated with respect to other important independent measures such as biopsies and post-procedural blood samples. These limitations have been due to the lack of relevant data.

In this study, a new modeling framework is created (Fig 1C) by combining i) state-of-the-art MRI processing of high resolution gadoxetate-enhanced time series [25, 26]; ii) the mechanistic gadoxetate uptake model, [23]; and iii) NLME model parametrization methods. To validate the model, a large clinical study was conducted by recruiting 100 patients, who were subjected to a variety of different measurements. These new data validate the model in three different ways. First, the extended model can describe patient variation across all stages of CLD. Second, the model can predict quantified images from later time points, which were not included in the estimation data; this implies the possibility of a shorter clinical protocol. Third, the model can predict independent validation data from blood samples and biopsies. Finally, it is demonstrated how the estimated model properties, such as OAPT1 and MRP2 transport rates, change with varying severity of CLD. These results point to a new avenue for estimation of liver function.

## Results

### Study population

A total of 100 patients, with suspected CLD were included in the study and each underwent an MRI examination followed by two liver biopsies. Of these, eight patients were excluded because they aborted the examination and one patient was excluded due to poor data quality, giving a final cohort of 91 patients. The demographic characteristics and clinical diagnoses of the final study population are presented in Table 1. None of the included patients showed signs of hepatic decompensation.

**Table 1:** Demographic and clinical data from of the final study population (N = 91).

	Median	Range
**Male (N)**	50	
**Age (Years)**	53	20–81
**BMI (kg/m**^**2**^**)**	26.4	16.9–35.0
**Bilirubin (μmol/L)**	11	4–48
**AST (μkat/L)**	0.75	0.29–4.50
**ALT (μkat /L)**	1.10	0.19–9.10
**ALP (μkat /L)**	1.20	0.43–10.00
**Fibrosis Stage**		
** F0**	29	
** F1**	16	
** F2**	25	
** F3**	14	
** F4**	7	
**Diagnosis**		
** Normal**	8	
** NAFLD**	35	
** HCV**	8	
** PSC**	13	
** PBC**	4	
** AIH**	12	
** AIH-PSC overlap**	2	
** AIH-PBC overlap**	1	
** Hemochromatosis**	1	
** DILI**	2	
** Wilson´s disease**	1	
** ALD**	2	
** AAT deficiency**	1	

*BMI* body mass index, *AST* aspertate aminotransferase, *ALT* alanine aminotransferase, *ALP* alkaline phosphatase, *NAFLD* non-alcoholic fatty liver disease, *HCV* hepatitis C virus infection, *PSC* primary sclerosing cholangitis, *PBC* primary biliary cirrhosis, *AIH* autoimmune hepatitis, *DILI* drug induced liver injury, *ALD* alcoholic liver disease, *AAT deficiency* α1-antitrypsin deficiency.

### The model framework is applicable to all stages of chronic liver disease

**Fig 2:**
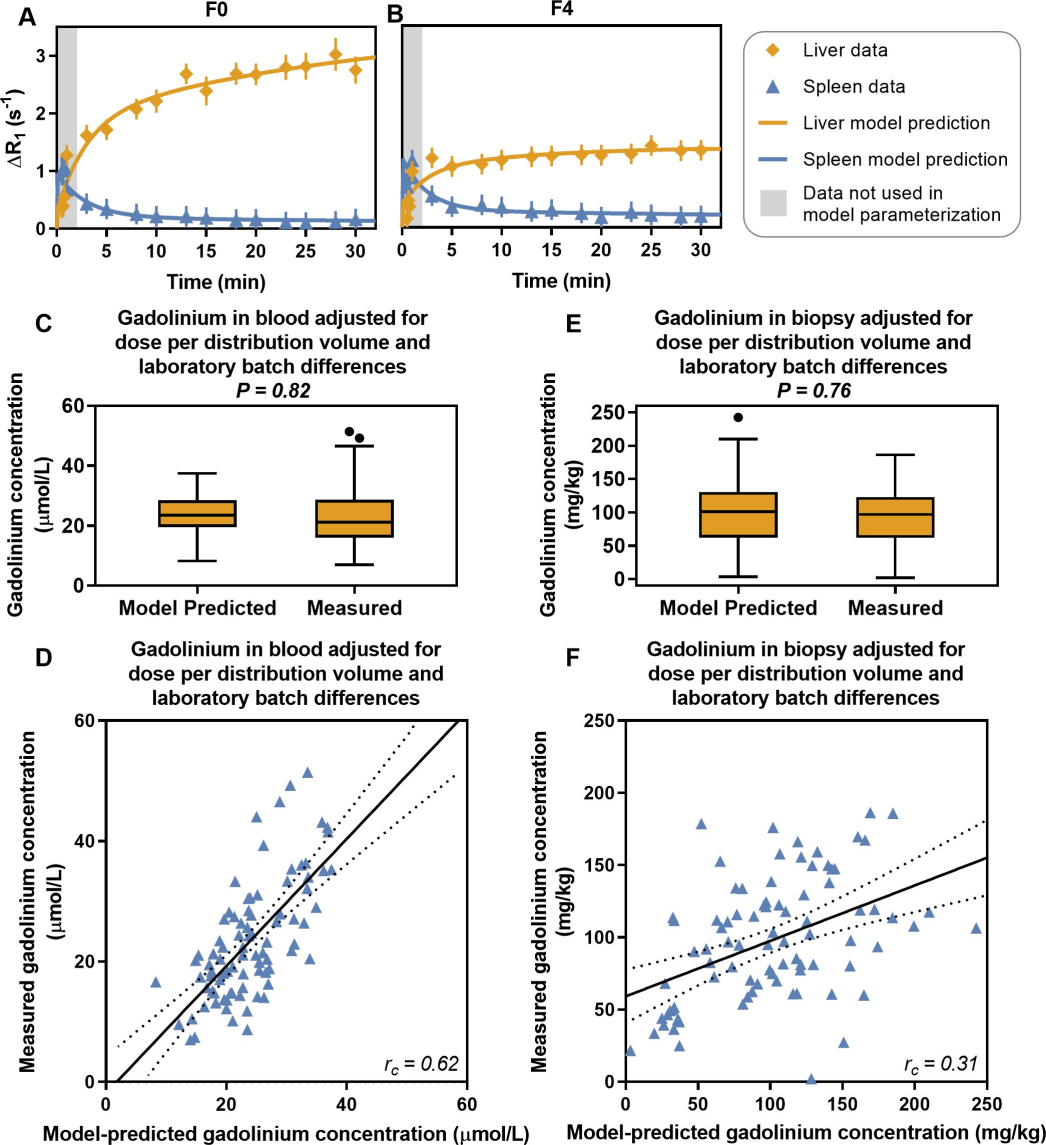
The mechanistic model framework predicts observed gadoxetate levels in chronic liver disease. (**A, B**) Observations and model-based predictions of gadoxetate levels, indicated by the changes in R_1_ relaxation rate (ΔR_1_, which is directly proportional to the gadoxetate concentration) in two patients, one with no fibrosis (F0) and one with histologically proven cirrhosis (F4). (**C, E**) The model is validated by predicting the gadoxetate concentration in blood and biopsy samples, which was acquired after the MRI examination. (**D, F**) Correlation between the measured and model-predicted gadolinium concentrations. The solid line is a linear regression and the dotted lines are 95% confidence intervals.

All patients were given an injection of gadoxetate and several MR-images were acquired over a period of 30 minutes for each patient. Time series for each patient were made by quantifying the change in R_1_ relaxation rate (ΔR_1_), which is proportional to gadoxetate concentration [27], in the liver and spleen. These time series were used to parametrize the mechanistic model (Fig 1D) for each individual patient using the NLME method. That is, the NLME algorithm was used to identify optimal model parameter values for each patient (e.g., describing the function of OATP1 and MRP2) such that the model predictions of the MRI data in the liver and the spleen matched the measured MRI data. Fig 2A and 2B shows the observed values and the model predictions for two typical patients, one without fibrosis (F0) and one with cirrhosis (F4). Goodness-of-fit for was assessed each patient. In all but one of the 91 patients, the model predicted the observed MRI data without being rejected by the goodness-of-fit test. This indicated that the same mechanisms were at play in all stages and etiologies of CLD, and that only the quantitative details were different.

### Measurements of gadoxetate concentrations in blood and biopsy samples validates the model

Fig 2C–2F shows a comparison between the gadolinium concentrations (the paramagnetic nucleus responsible for the contrast enhancement in gadoxetate) measured in the blood samples and biopsies and the gadolinium concentrations predicted by the mathematical model. At a group level, there were no differences between the gadolinium concentrations predicted by the mathematical model and the concentrations measured using inductively coupled plasma sector field mass spectrometry (ICP-SFMS) (Fig 2C and 2E). At an individual level, there was a moderate Lin’s concordance correlation between the predicted and measured gadoxetate concentrations in blood samples (r_c_ = 0.62; Fig 2D). However, there was only a low correlation in liver biopsy samples (r_c_ = 0.31; Fig 2F).

### NLME enables short-duration gadoxetate MRI examinations

To assess whether the NLME-model parameterization method outperforms the standard two-stage approach (STS), and whether it is possible to reduce the examination time to 10 min from gadoxetate injection, the dataset for each patient was divided into two parts: all data points from within 10 min after gadoxetate injection were used as estimation data, and the validation data included the remaining later time points. Both the NLME and STS parametrization methods were used to parametrize the model with the estimation data. The resulting model predictions were compared to the estimation data and validation data, and goodness-of-fit was assessed for each patient individually.

The NLME parameterization was implemented using a 'leave-one-out' design, meaning that one data set at a time was truncated while all other data sets were complete. This design was to demonstrate how NLME could be used in a clinical situation where the distributions of the parameters have already been determined in clinical studies. Four patients had insufficient data for this analysis (there was no available data after 10 min), so the test included data from 87 patients.

**Fig 3:**
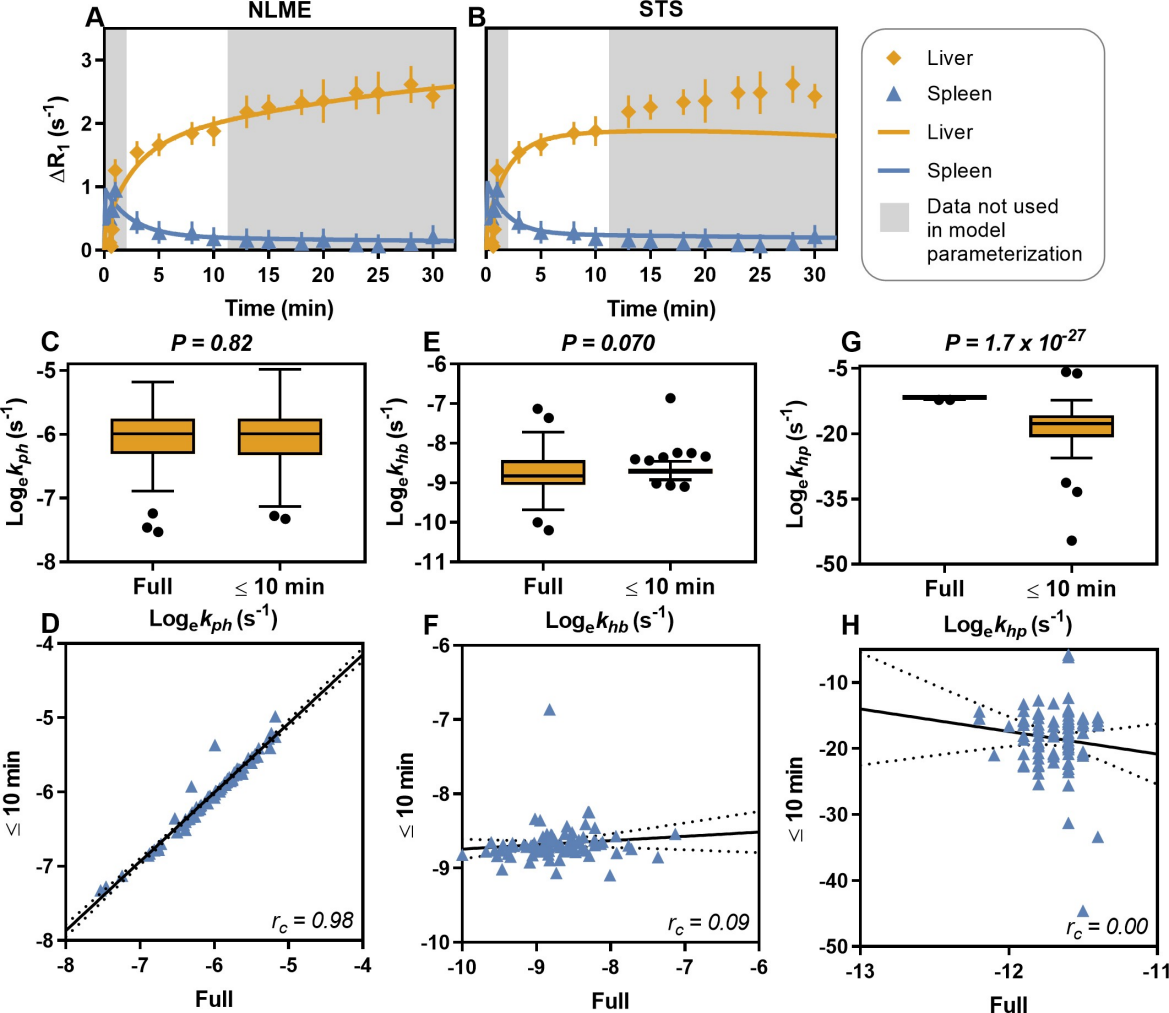
The non-linear mixed effects (NLME) model parametrization enables shorter gadoxetate MRI examinations. The models were parametrized with data up to 10 min after gadoxetate injection and were validated against the remaining data. (**A**) shows an example of simulation after NMLE parameterization, (**B**) shows a simulation of the same patient after STS parameterization. The natural logarithms of the model parameter values hepatocyte uptake rate (k_ph_; **C-D**), hepatocyte elimination rate (k_hb_; **E-F**), and hepatocyte to plasma flux (k_hp_; **G-H**), estimated with the NLME method using the full data set, were compared with the parametrization using data up to 10 minutes. Significant differences were observed for k_hp_. The solid line is a linear regression and the dotted lines are 95% confidence intervals.

Both the NLME and STS methods produced model predictions that passed the statistical test for goodness-of-fit for the estimation data in all 87 patients. The NLME method predicted the data in the validation dataset in 81 patients (93%) without being rejected, compared with 37 patients (43%) with the STS method. Fig 3A and 3B shows an example of a patient for whom the NLME method could predict the validation data, while the STS method failed, particularly when predicting the liver signal.

Significant Lin’s concordance correlation was observed between predicted and measured blood plasma gadoxetate concentrations when using the NLME parametrization to data from the first 10 min (r_c_ = 0.60). Notably, there was no significant difference between the predicted and measured blood plasma concentrations with prediction based on parametrization to data from the first 10 min. Correlation between predicted and measured blood plasma gadoxetate concentrations based on the STS parametrization was not as strong as with the NLME parametrization (r_c_ = 0.24).

The array of model parameters for each patient, estimated by the NLME parametrization method using data from the first 10 min, were compared with the same parameters estimated by the NLME parametrization method using the full dataset (Fig 3C–3H). The OATP function, i.e. hepatocyte uptake rate (*k*_*ph*_; Fig 3C), was unaffected by the sparse (≤10 min) estimation with a highly significant correlation between sparse and full estimations (Fig 3D). At a group level, the MRP2 function, *i*.*e*. hepatocyte elimination rate (*k*_*hb*_; Fig 3E), was unaffected by the sparse estimation data. However, the individual values for each patient were affected and hence the correlation was poor (Fig 3F). The hepatocyte to plasma flux (*k*_*hp*_; Fig 3G) was significantly affected by the sparse estimation data, with a non-significant correlation (Fig 3H).

### Hepatic accumulation of gadoxetate is significantly affected in patients with fibrosis

**Fig 4:**
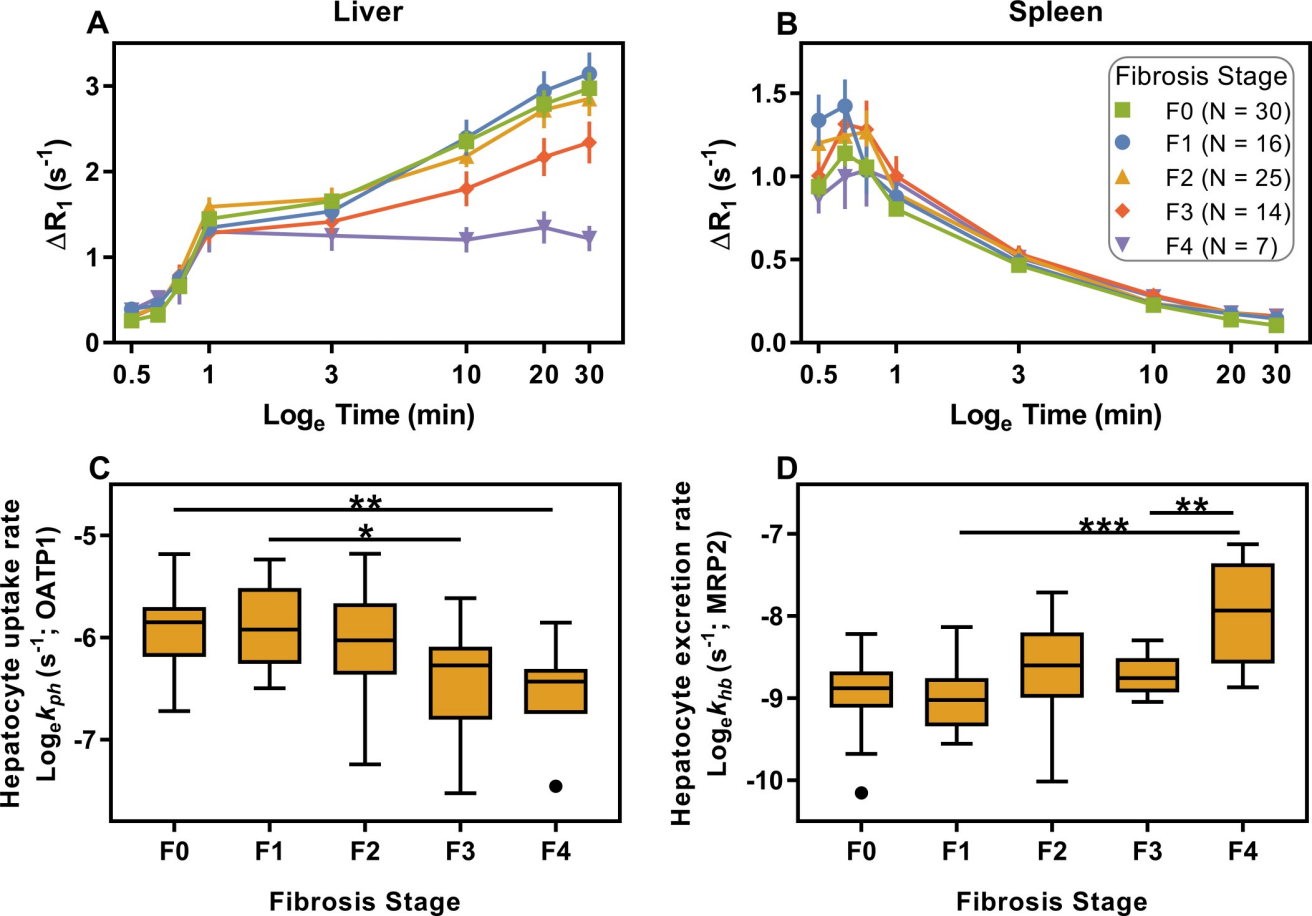
Effects of fibrosis on hepatic function. Liver (**A**) and spleen (**B**) time series showing average induced change in R_1_ relaxation rate (ΔR_1_) in patients with different levels of fibrosis. Error bars indicate the standard error of the mean. In (**C, D**), liver function parameters are shown for each level of fibrosis. Horizontal lines indicate significant differences (ANOVA, Tukey’s post-test: * <0.05; ** <0.01; *** <0.001).

In the liver, ΔR_1_ is lower in patients with increased fibrosis stage (Fig 4A). In the spleen, ΔR_1_ appears to be unaffected (Fig 4B). Furthermore, the hepatocyte uptake rate of gadoxetate by OATP1, differentiated between fibrosis stages, is shown in Fig 4C. The figure shows that the uptake is decreased in patients with advanced fibrosis and cirrhosis. Furthermore, the hepatocyte excretion rates were differentiated between cirrhosis and both advanced fibrosis and mild fibrosis (Fig 4D). Finally, Table 2 shows a confusion matrix of the ability of *k*_*ph*_ to identify patients with advanced fibrosis, i.e. ≥F3, when using a cut-off of 0.00198 s^-1^.

**Table 2:** Confusion matrix for the ability of *kph* to identify patients with advanced fibrosis.

	Predicted No Advanced Fibrosis	Predicted Advanced Fibrosis
**True No** **Advanced Fibrosis**	53	17
**True** **Advanced Fibrosis**	5	16

## Discussion

A new next-generation framework to measure liver function using MRI was developed. This framework was successfully applied and validated with liver biopsy and blood samples in a clinical setting in a diverse cohort. More specifically, the model could describe data from all patients and it was able to adequately predict gadoxetate levels in both blood plasma and biopsies. Furthermore, the introduced NLME method for parameter estimation is more robust on shorter protocols, compared to the previously used STS method; this allows for shorter examinations. Finally, the validated model allowed for the examination of how the biomedical mechanisms for clearance-based liver function vary across different stages of CLD.

The new framework is validated in several different ways. First, the model was validated by the fact that it could be used to extrapolate data points not used for parameter estimation (example in Fig 3A and 3B). Second, the model could also be fitted to data from patients with a wide variety of different chronic liver diseases (Table 1). Third, the concentrations of gadoxetate in *both* liver biopsy and blood samples were measured by ICP-SFMS (Fig 2C–2F). On a group level, there was no significant difference between the predicted and measured gadoxetate concentrations. On an individual level, there was a moderate correlation in the blood, while there was a low individual correlation in the biopsy. This lower correlation may be due to contamination of the biopsy samples from gadoxetate in the bile ducts. These correlations do not necessarily mean that our modeling framework should be used for individual predictions of gadoxetate concentrations. However, the results do show that our modeling framework produces realistic parameter values. Last, the framework was also validated by the fact that the model parameters corresponding to OATP1 and MRP2 functions varied as expected in the patient population (Fig 4).

The variation of the parameters across the patient population requires some additional remarks. First, the population covered a wide spectrum of both etiologies (Table 1) and severity and we used liver fibrosis to indicate severity. Second, hepatic uptake via OATP1 transporters (*k*_*ph*_) decreased significantly with increasing levels of fibrosis. Similar results were previously obtained in studies with perfusion-style model frameworks [28, 29]. Possible reasons for reduction of OATP1 function include restricted access of gadoxetate to the hepatocytes, reduction in the number of functional hepatocytes, and competitive inhibition. Third, with respect to hepatic excretion via MRP2 (*k*_*hb*_), a significantly higher excretion rate was estimated in patients with cirrhosis, compared to patients with lower levels of fibrosis. Other studies have reported mixed results. Previously, a small study using a perfusion model indicated the opposite, that gadoxetate excretion decrease in cirrhotic humans [30]. Therefore, it is interesting to look at studies of gene expression. Some studies on cirrhotic rats have shown an upregulation of MRP2 [31–33], which is consistent with our findings. In contrast, one study found a lower expression of MRP2 in rats with fibrosis [34]. In humans, the picture is also mixed and CLD has been found to be associated with either no difference [35], a slight increase [36], or in some CLD etiologies, a decrease [37] in MRP2 expression.

By using the NLME parameterization scheme, the time needed for MRI-examinations could be reduced, while still being able to estimate reliable parameters, as well as predicting both the liver and spleen signals (Fig 3). This reduction was accomplished because NLME allows for information to be shared among the parameter estimations of all patients, thus requiring fewer new datapoints per patient. This reduction in the examination time is beneficial, since it reduces cost and patient discomfort, and requiring only a few images also allows for our method to be included for liver function evaluation in short abbreviated MRI-protocols. Such protocols, (sometimes called AMRI) are gaining popularity, e.g. when screening cirrhotic patients for hepatocellular carcinoma [38, 39].

It can be noted that while the STS scheme failed to predict the liver signal, STS was still able to predict the spleen signal. The reason for this is that almost all dynamic information of the spleen signal is contained within the first ten minutes. Removing all later time points should therefore not affect the ability to predict the spleen signal. Furthermore, it can also be noted that while the χ^2^-test was used to evaluate the goodness-of-fit of the model, the NLME framework offers other methods for assessing model performance, such as visual predictive check and normal predictive distribution errors. However, since NLME was only used to increase the robustness of the predictions of the individual patients, the χ^2^-test should be enough. Comparing different methods would be interesting, but was unfortunately beyond the scope of this work.

Another strength of this work is that data are presented from a new clinical study, where 100 patients were recruited. The patients were selected to represent the actual flow of patients being referred to a hepatology department, with a normal variation in both disease etiology and severity. This gives a more realistic picture of the clinical situation, as most other studies have either been small or not prospective. Additionally, the study included dual biopsies, as well as blood samples, from the patients, in conjunction with the MR examination. These rare measurements allowed for extensively validate the model. Lastly, the study was conducted over a span of around six years. Such a long time could be seen as a limitation, as changes occur to an MR-system over time, such as software upgrades. On the other hand, this could also be seen as a strength of the method, since it was found that all data could easily be analysed in the same framework.

Although this methodology is still in the research phase, the methodology is better suited for clinical implementation, compared to other similar methods, for a variety of reasons. First, the modeling framework uses the same type of clinical images, already collected in routine examinations. Therefore, the liver function estimation can easily be included in clinical workflows or studies that already use gadoxetate MRI, by simply adding a few more breath holds. Second, the model is based on simulations of ordinary differential equations, which has additional advantages. For instance, the model, unlike previous non-simulation-based models [19, 21, 22], can easily be combined with other models describing detailed processes in the liver, and thus can possibly characterize other aspects of liver function, such as metabolic aspects [40]. Third, the simulation-driven model can also be combined with more zoomed out whole-body models. The result of such combinations is multi-level models which can simultaneously describe multiple organs and processes in the body [41–43]. For all these reasons, the framework could be further extended and reused in a variety of different contexts, both regarding clinical implementation and research.

In conclusion, this study presented a new integrative MRI-based framework for estimating liver function. The extendable framework has been validated in a variety of ways and has allowed for a new and deeper look into the variation of mechanistic parameters across a clinically relevant cohort.

## Methods

### Study design and population

Between 2007 and 2014, 100 patients were recruited on referral to the Linköping University Hospital, Linköping, Sweden for evaluation of chronic (> 6 months) elevation of levels of one or more of ALT (>1.10 μkat/L for men and >0.75 μkat /L for women), aspartate aminotransferase (AST; >0.75 μkat /L for men and >0.60 μkat /L for women), and serum alkaline phosphatase (ALP; >1.80 μkat /L regardless of gender). All patients who, on clinical indication or as part of a clinical study, needed a liver biopsy for histopathological evaluation were asked to participate in the study. Exclusion criteria included contraindications for MRI (presence of pacemaker devices, implants with ferromagnetic properties, pregnancy, and claustrophobia) and liver biopsy (presence of primary or secondary coagulative disorder, prothrombin time > 1.5 times the international normalized ratio, platelet count <50×10^9^ /L, hepatic malignancy, and clinical signs of decompensated cirrhosis).

A diagnostic work-up was performed prior to MRI, including a physical examination, laboratory investigations, and ultrasonography. The pathologist was blinded to the results of the diagnostic work-up, and the radiologists were blinded to the diagnosis and the pathology findings.

### Ethics statement

This study was approved by the regional ethics committee (Reference No. M72-07 T5-08). All patients gave informed consent to participate before the inclusion.

### Gadoxetate-enhanced magnetic resonance imaging

MRI was performed within two months of the diagnostic work-up with a Philips Achieva 1.5 T MR scanner (Philips Healthcare, Best, Netherlands) and a phased-array body coil. Single-breath-hold symmetrically sampled T1-weighted gradient-echo two-point Dixon 3D images [44] were acquired using sensitivity encoding [45].

**Fig 5:**
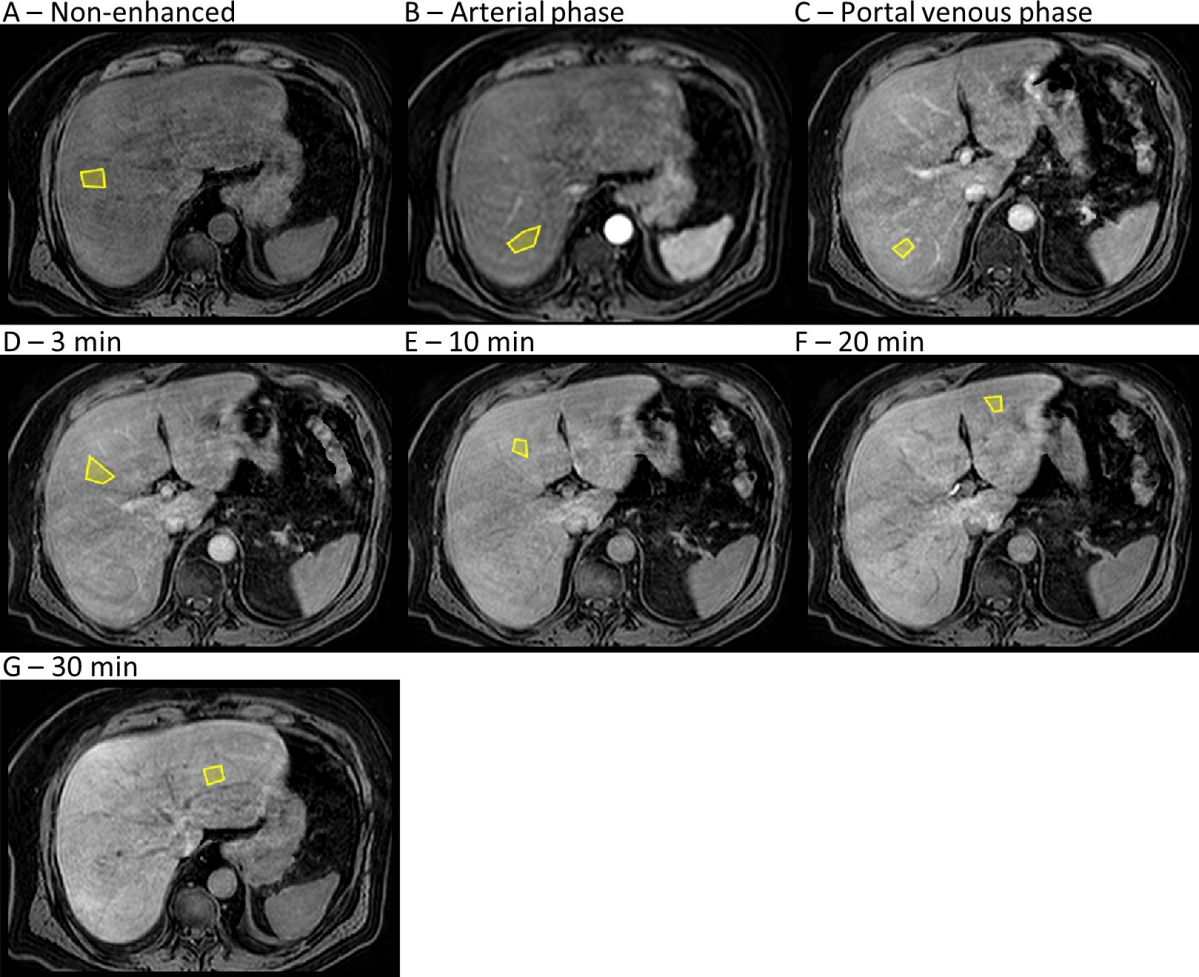
Gadoxetate MRI time series of the liver. (**A**-**G**) Representative placement of seven regions of interest (yellow polygons) within the liver, of which four (**A**-**D**) were placed in the right liver lobe and three (**E**-**G**) were placed in the left liver lobe. This set shows an entire time series in a single patient, from before gadoxetate injection (**A**) to 30 min after gadoxetate injection (**G**). The arterial phase (**B**) typically occurs 30 s after gadoxetate injection, and the portal-venous phase (**C**) typically occurs 1 min after gadoxetate injection.

All patients received a bolus injection of gadoxetate (gadolinium ethoxybenzyl diethylenetriamine pentaacetic acid, or Gd-EOB-DTPA, marketed as Primovist in Europe and Eovist in the USA by Bayer Schering Pharma, Berlin, Germany), at a dose of 0.1 ml/kg, administered intravenously at a rate of 1 mL/s by a power injector (Medrad Spectris Solaris, Pittsburgh, PA, USA), followed by a 30 mL saline flush. Image time series were acquired prior to (non-enhanced) and directly following gadoxetate injection (Fig 5). The post-injection time series corresponded to the arterial and portal-venous phases, as well as 3 min, 10 min, 20 min, and 30 min following injection. Additional acquisitions between 3 min and 30 min were added from 2012 and onwards.

The field of view (FOV) and acquisition matrix were adjusted to accommodate patients of different sizes. Higher temporal resolution was used during the initial contrast agent wash-in phase, the arterial phase. The non-enhanced and post-injection images were acquired using the following sequence parameters: repetition time = 6.5 ms, echo time = 2.3 ms and 4.6 ms, flip angle = 13°, typical acquisition matrix = 168×168, typical FOV = 261 mm by 200 mm by 342 mm, slice thickness = 4 mm. We used interpolation, with zero padding in the z-direction, and up-interpolated from 4 to 2 mm.

### Image post-processing

The acquired in-phase and opposite-phase images were reconstructed into separate water and fat images by our previously developed inverse-gradient method [26]. The signal intensity of MR-images is not absolute. Hence, if images are acquired in a time series, the signal intensity in the images can vary, even though all images are acquired using the same parameters. We corrected for this variation by using voxels of pure adipose tissue as an internal reference throughout the time series. [25] This was an important step in the quantification process.

To extract signal intensities (SIs) for the quantitative analysis, two clinical radiologists (BN, ND; with more than ten years of experience in abdominal radiology) placed ROIs in the reconstructed water-image time series, in the liver (*N* = 7), and spleen (*N* = 3). Liver ROIs were placed in both the left and right liver lobes to avoid any large vessels or focal lesions, but not strictly following the Couinaud segmental division. The sizes of ROIs were arbitrarily chosen by the radiologists. However, the ROIs were adjusted to be equal in size and approximate position throughout the time series. Landmarks in the images were used to correct for movement of the liver between the acquisitions. Fig 5 shows an example of ROI placement.

Mean SIs in the ROIs were normalized and the relaxation rate (R_1_) was calculated as described previously [46]. The induced change in the relaxation rate (ΔR1) was directly proportional to gadoxetate concentrations [27].

For quality assurance, the image data were inspected visually for quality issues and potential data exclusion. As the two radiologists independently placed ROIs in the images, they took particular notice of cases of poor image quality (such as artifacts resulting from breathing or post-processing failure). Then, both radiologists reviewed these cases of potential poor quality and reached a consensus about whether to exclude the images, to return the images for manual image reconstruction, or to accept the images. After the radiologists were satisfied, the data analyst continued to search for any significant outliers in the extracted time series. The radiologists were then instructed to review these latter outliers, but they were not told why each case was to be reviewed. If they were still satisfied with the placement of ROIs and with the image quality, nothing was corrected.

### Estimation of the lower limit of data uncertainty

The lower limit of uncertainty in the extracted gadoxetate time series was estimated by calculating the average standard deviation in the normalized signal intensities (Eq 1, where S = SI(t)/SI(t = 0)).

$$\sigma S=|S|\sqrt{{\left(\frac{\sigma SI(t)}{SI(t)}\right)}^{2}+{\left(\frac{\sigma SI(t=0)}{SI(t=0)}\right)}^{2}}$$ (1)

**Fig 6:**
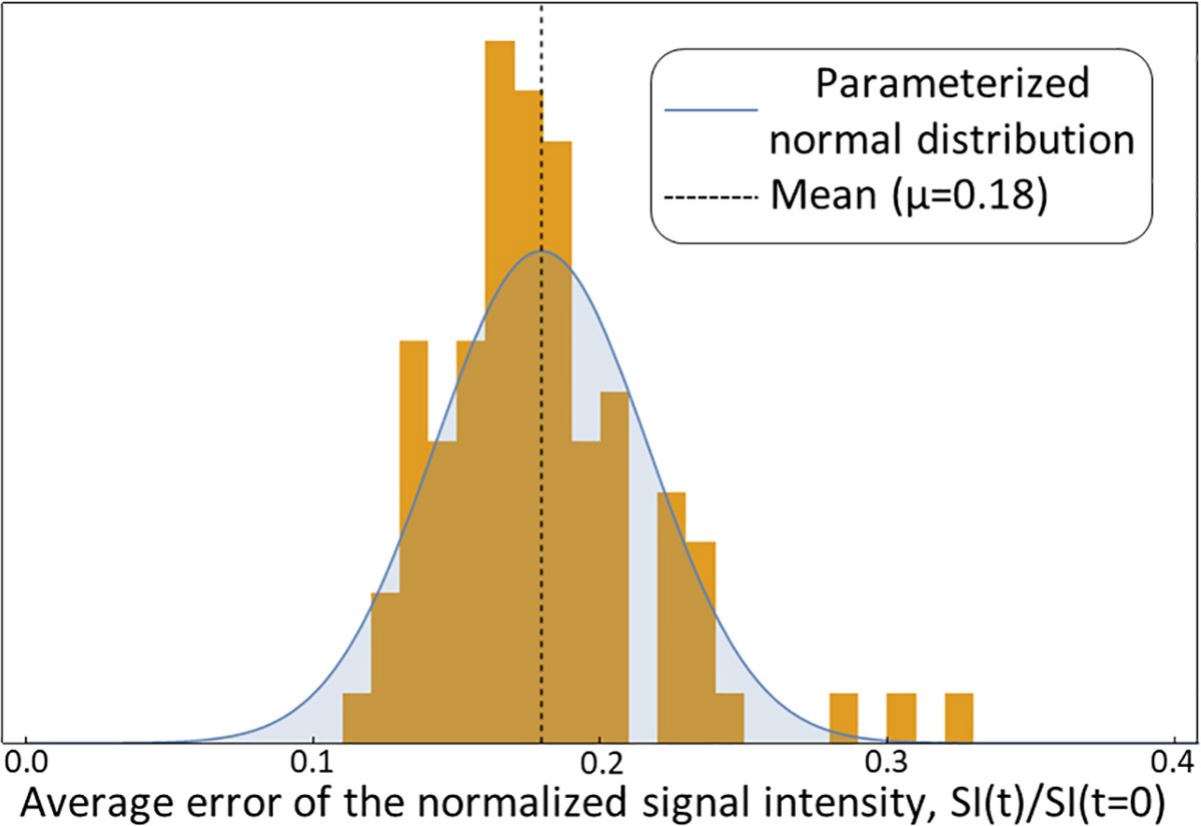
Estimation of the lower limit of data uncertainty. Histogram of the average uncertainty of the normalized signal intensity in the liver and spleen regions of interest, with one entry per patient. The blue bell curve shows the fitted normal distribution, which indicates a lower limit of uncertainty of 0.18.

The uncertainty was then averaged over the entire study population; for this averaging, each entry was in turn the mean of each patient’s liver and spleen ROIs. Fig 6 shows a histogram of the estimate of the lower limit of uncertainty, and the fitted normal distribution had a mean of 0.18. Unless the standard error of the mean across the spleen or liver ROIs exceeded 0.18, this lower limit value was used as the standard deviation in the following statistical test for the mechanistic model goodness-of-fit.

### Mathematical model and model parametrization

The whole-body model devised by Forsgren and co-workers [23] was used here to quantify the liver function. Fig 1C shows a schematic diagram of the model, which has two parts: a dynamic model and a signal model. Briefly, the dynamic model describes five separate fluxes of gadoxetate: between the blood plasma and the extracellular extravascular space (EES; *k*_*diff*_); elimination via the kidneys to urine (*CLr*); uptake into the hepatocytes (through the OATP1 family transport proteins; *k*_*ph*_); back-flux from the hepatocytes into the blood plasma (through the transport protein MRP3; *k*_*hp*_); and excretion from the hepatocytes into the bile (through the transport protein MRP2; *k*_*hb*_):

$$\frac{{dC}_{hep}}{dt}=k_{ph}C_{P}Alb-k_{hp}C_{hep}-k_{hb}C_{hep},$$ (2)

$$\frac{dC_{p}}{dt}=\frac{(k_{hp}C_{hep}-k_{ph}C_{P}Alb)V_{l}v_{h}-CLrC_{P}Alb+(k_{diff}C_{ees}-k_{diff}C_{P}Alb)V_{ees}+u}{V_{p}},$$ (3)

$$\frac{{dC}_{ees}}{dt}=k_{diff}C_{P}Alb-k_{diff}C_{ees},$$ (4)

where C_hep_, C_p_, and C_ees_ is the gadoxetate concentration in the hepatocytes, blood plasma, and EES respectively. V_l_, V_ees_, and V_p_ are the volumes of the liver, EES, and blood plasma respectively (assumed to be 1.43, 14.77 and 2.57 L). Alb is the fraction of Gadoxetate not bound to serum albumin (assumed to be 0.9), v_h_ is the volume fraction of hepatocytes in the liver (assumed to be 0.68), and u is the injection of gadoxetate. CLr is assumed to be 118 mL/min.

The signal model was used to predict ΔR_1_ in the gadoxetate MRI time series as a function of the gadoxetate concentrations in the compartments. The model takes into account the parenchyma volume fractions as well as the *in situ* tissue-specific relaxivity properties of gadoxetate [23]:

$${\Delta R}_{1,l}=\xi (C_{hep}v_{h}r_{1,hep}+C_{p}v_{p,l}r_{1,p}+C_{ees}v_{ees,l}r_{1,ees}),$$ (5)

$${\Delta R}_{1,s}=\xi (C_{p}v_{p,s}r_{1,p}+C_{ees}v_{ees,s}r_{1,ees}),$$ (6)

where ΔR_1,l_ and ΔR_1,s_ are the ΔR_1_ in the liver and spleen respectively, v_p,l_ and v_ees,l_ are the volume fractions of plasma and EES in the liver (assumed to be 0.12 and 0.20), and v_p,s_ and v_ees,s_ are the fractions of plasma and EES (assumed to be 0.35 and 0.20) in the spleen. ξ is an arbitrary scaling parameter and r_1,hep_, r_1,p_, and r_1,ees_ are the tissue-specific relaxivities in the hepatocytes, blood plasma, and EES respectively (assumed to be 10.7, 7.3, and 6.9 mmol^-1^s^-1^).

The model was parametrized separately using the STS method and the NLME method, described in Fig 1A and 1B. The STS parametrization was performed by minimizing the following costfunction, which follows a χ^2^-distribution:

$$V(\hat{p})=\sum \frac{{\left({\hat{y}}_{i}(\hat{p},t)-y_{i}(t)\right)}^{2}}{{\sigma^{2}}_{i}(t)}\in \chi^{2}(df),$$ (7)

where y and σ are the measurements and standard deviation of the measurements respectively, $\hat{y}$ is the predicted data as a function of time and the estimated model parameters ($\hat{p}$), and the index *i* indicates liver or spleen.

When using NLME, all the optimized parameters have two parts, a fixed effect and a random effect. The fixed effect is the same across all patients and represents the typical parameter value. The random effect describes how each individual deviate from the typical value and is thus allowed to vary across the population, but is still constrained to a normal distribution:

$$p^{j}=\theta_{p}+\eta_{p}^{j}$$ (8)

$$p^{j}=\theta_{p}e^{\eta_{p}^{j}}$$ (9)

where p^j^ is a generic parameter for patient j, θ_p_ is the fixed effect for parameter p, and η^j^_p_ is the random effect for parameter p for patient j. If p is postulated to be normally distributed, Eq 8 is used, while Eq 9 is used if p is lognormally distributed. More details of the STS parametrization are described in [23] and the details of NLME in [24, 47].

Population distributions in the NLME model parametrization were defined as a normal distribution for the scale parameter (ξ) and lognormal distributions for the four rate parameters (*k*_*diff*_, *k*_*ph*_, *k*_*hp*_, and *k*_*hb*_). The distributions were *a priori* parametrized from the results of parametrizing the model to healthy human patients, which has been described previously (Table 3 in [23]), where the expectation values were ξ = 1.6, *k*_*diff*_ = 1.7 ms^-1^, *k*_*ph*_ = 4.7 ms^-1^, *k*_*hp*_ = 28 ms^-1^, and *k*_*hb*_ = 38 ms^-1^. The *a priori* standard deviations were chosen such that the optimization algorithm would not be unnecessarily limited (ξ = 1, *k*_*diff*_ = 0.1 ms^-1^, *k*_*ph*_ = 0.1 ms^-1^, *k*_*hp*_ = 0.01 ms^-1^, and *k*_*hb*_ = 0.01 ms^-1^).

The mechanistic model framework assumes that the compartments are well mixed containers (a fundamental property of ordinary differential equation models). In addition, there are interfering effects from the bolus injection during the arterial and portal-venous phases. Therefore, only data at the 3 min point and later after contrast injection were included in the model parameterization.

### Blood sampling and elemental analysis

Immediately following the MR examination, 3 mL venous blood (collected in a 3 mL BD Vacutainer sterile hematology tube with K_2_-EDTA) was drawn from each patient for elemental analysis of gadolinium content. Samples were transferred to 4 mL sterile low-temperature freezer vials (VWR, Sweden) for freezing and storage at -80°C. The frozen samples were sent to an external laboratory (ALS Scandinavia AB, Luleå, Sweden) for elemental analysis by ICP-SFMS: 0.20 mL from each thawed blood sample was mixed with 1.00 mL ‘super pure’ HNO_3_ (pure with respect to traces of metal) and digested in a 600 W microwave oven operating at 75% power for 30 min. Each of these mixtures was then diluted up to 10.00 mL with MilliQ ultrapure water for ICP-SFMS analysis, which had a detection limit for gadolinium of 0.05 μg/L.

### Liver biopsy and histopathology

Immediately after completion of the MR examination and blood sampling, two ultrasonographically guided liver biopsy procedures were performed, on an outpatient basis. The biopsy samples were obtained percutaneously with a 1.6 mm BioPince needle (BioPince Full Core Biopsy Instrument, Argon Medical Devices, Plano, TX, USA) in either the left or right liver lobe depending on which location offered the best combination of a successful biopsy and maximum patient safety. A histopathologist graded and classified one of the biopsy samples according to the Batts and Ludwig system [48], through which fibrosis was staged as no fibrosis (F0), portal and/or perisinusoidal fibrosis (F1), periportal and perisinusoidal fibrosis (F2), bridging fibrosis (F3), and probable or obvious cirrhosis (F4). The biopsies were also graded for inflammation. All biopsy samples were graded by the same histopathologist.

The second biopsy sample of each pair was weighed and directly frozen at -80°C. The frozen samples were later freeze dried, and the dry weight was measured prior to submission to our external analysis partner (ALS Scandinavia AB) for elemental analysis. The dried samples were digested by adding 2.50 mL ‘super pure’ HNO_3_ and 0.25 mL H_2_O_2_ followed by a 30 min treatment at 170°C in a microwave oven. The samples were then diluted to 5.00 mL with MilliQ ultrapure water, for ICP-SFMS analysis.

### Statistical analysis

The goodness-of-fit of the model to the data was investigated on a subject basis using a χ^2^ test (Eq 7; α = 0.05) with degrees of freedom equal to the number of observations in the gadoxetate-enhanced MRI time series. Group differences were investigated using an unpaired two-tailed Mann–Whitney U-test (α = 0.05). A paired two-tailed Mann–Whitney U-test was used when comparing two model parametrization method estimates of model parameters (α = 0.05). Linear regression and Lin’s concordance correlation were used to investigate correlation between variables that measure or describe similar entities. For correlation between non-similar variables, a Pearson correlation coefficient was calculated. An ANOVA with Tukey’s post-test was used to investigate sources of variation and biomarker performance between fibrosis stages.

## Supporting information

S1 File Matlab data. The file contains the time series for each patient together with the fibrosis stage, saved in .mat format.
(MAT)

## References

[1] Manizate F, Hiotis SP, Labow D, Roayaie S, Schwartz M. Liver functional reserve estimation: state of the art and relevance for local treatments. J Hepatobiliary Pancreat Sci. 2010;17(4):385–8. 10.1007/s00534-009-0228-x 19936599

[2] Etra JW, Squires MH, Fisher SB, Rutz DR, Martin BM, Kooby DA, et al Early identification of patients at increased risk for hepatic insufficiency, complications and mortality after major hepatectomy. HPB: the official journal of the International Hepato Pancreato Biliary Association. 2014;16(10):875–83. 10.1111/hpb.12270 24836954 PMC4238853

[3] Navarro VJ, Senior JR. Drug-related hepatotoxicity. N Engl J Med. 2006;354(7):731–9. 10.1056/NEJMra052270 16481640

[4] MacDonald JS, Robertson RT. Toxicity testing in the 21st century: a view from the pharmaceutical industry. Toxicol Sci. 2009;110(1):40–6. 10.1093/toxsci/kfp088 19435982

[5] Senior JR. Alanine aminotransferase: a clinical and regulatory tool for detecting liver injury-past, present, and future. Clinical pharmacology and therapeutics. 2012;92(3):332–9. Epub 2012/08/09. 10.1038/clpt.2012.108 22871997

[6] Amacher DE, Schomaker SJ, Aubrecht J. Development of blood biomarkers for drug-induced liver injury: an evaluation of their potential for risk assessment and diagnostics. Mol Diagn Ther. 2013;17(6):343–54. 10.1007/s40291-013-0049-0 23868512

[7] Kanaya N, Iwasaki H, Namiki A. Noninvasive ICG clearance test for estimating hepatic blood flow during halothane and isoflurane anaesthesia. Can J Anaesth. 1995;42(3):209–12. 10.1007/BF03010678 7743571

[8] Nishie A, Ushijima Y, Tajima T, Asayama Y, Ishigami K, Kakihara D, et al Quantitative analysis of liver function using superparamagnetic iron oxide- and Gd-EOB-DTPA-enhanced MRI: comparison with Technetium-99m galactosyl serum albumin scintigraphy. Eur J Radiol. 2012;81(6):1100–4. 10.1016/j.ejrad.2011.02.053 21435811

[9] Giannini EG, Fasoli A, Borro P, Botta F, Malfatti F, Fumagalli A, et al 13 C-galactose breath test and 13 C-aminopyrine breath test for the study of liver function in chronic liver disease. Clin Gastroenterol Hepatol. 2005;3(3):279–85. 10.1016/S1542-3565(04)00720-7 15765448

[10] Stieger B, Heger M, de Graaf W, Paumgartner G, van Gulik T. The emerging role of transport systems in liver function tests. Eur J Pharmacol. 2012;675(1–3):1–5. 10.1016/j.ejphar.2011.11.048 22173125

[11] Giacomini KM, Huang SM, Tweedie DJ, Benet LZ, Brouwer KL, Chu X, et al Membrane transporters in drug development. Nat Rev Drug Discov. 2010;9(3):215–36. 10.1038/nrd3028 20190787 PMC3326076

[12] Van Beers BE, Pastor CM, Hussain HK. Primovist, Eovist: what to expect? J Hepatol. 2012;57(2):421–9. 10.1016/j.jhep.2012.01.031 22504332

[13] Leonhardt M, Keiser M, Oswald S, Kühn J, Jia J, Grube M, et al Hepatic uptake of the magnetic resonance imaging contrast agent Gd-EOB-DTPA: role of human organic anion transporters. Drug metabolism and disposition: the biological fate of chemicals. 2010;38(7):1024–8. 10.1124/dmd.110.032862 20406852

[14] Pascolo L, Petrovic S, Cupelli F, Bruschi CV, Anelli PL, Lorusso V, et al Abc protein transport of MRI contrast agents in canalicular rat liver plasma vesicles and yeast vacuoles. Biochem Biophys Res Commun. 2001;282(1):60–6. 10.1006/bbrc.2001.4318 11263971

[15] Trauner M, Boyer JL. Bile salt transporters: molecular characterization, function, and regulation. Physiol Rev. 2003;83(2):633–71. 10.1152/physrev.00027.2002 12663868

[16] Motosugi U, Ichikawa T, Sou H, Sano K, Tominaga L, Kitamura T, et al Liver parenchymal enhancement of hepatocyte‐phase images in Gd‐EOB‐DTPA‐enhanced MR imaging: Which biological markers of the liver function affect the enhancement? J Magn Reson Imaging. 2009;30(5):1042–6. 10.1002/jmri.21956 19856436

[17] Asenbaum U, Kaczirek K, Ba-Ssalamah A, Ringl H, Schwarz C, Waneck F, et al Post-hepatectomy liver failure after major hepatic surgery: not only size matters. Eur Radiol. 2018;28(11):4748–56. 10.1007/s00330-018-5487-y 29767320 PMC6182758

[18] Schulze J, Lenzen H, Hinrichs JB, Ringe B, Manns MP, Wacker F, et al An Imaging Biomarker for Assessing Hepatic Function in Patients with Primary Sclerosing Cholangitis. Clin Gastroenterol Hepatol. 2018;17(1):192–9. 10.1016/j.cgh.2018.05.011 29775791

[19] Ulloa JL, Stahl S, Yates J, Woodhouse N, Kenna JG, Jones HB, et al Assessment of gadoxetate DCE‐MRI as a biomarker of hepatobiliary transporter inhibition. NMR Biomed. 2013;26(10):1258–70. 10.1002/nbm.2946 23564602 PMC3817526

[20] Karageorgis A, Lenhard SC, Yerby B, Forsgren MF, Liachenko S, Johansson E, et al A multi-center preclinical study of gadoxetate DCE-MRI in rats as a biomarker of drug induced inhibition of liver transporter function. PLoS One. 2018;13(5):e0197213 10.1371/journal.pone.0197213 29771932 PMC5957399

[21] Sourbron S, Sommer WH, Reiser MF, Zech CJ. Combined quantification of liver perfusion and function with dynamic gadoxetic acid–enhanced MR imaging. Radiology. 2012;263(3):874–83. 10.1148/radiol.12110337 22623698

[22] Georgiou L, Penny J, Nicholls G, Woodhouse N, Ble FX, Hubbard Cristinacce PL, et al Quantitative Assessment of Liver Function Using Gadoxetate-Enhanced Magnetic Resonance Imaging: Monitoring Transporter-Mediated Processes in Healthy Volunteers. Invest Radiol. 2016;52(2):111–9. 10.1097/rli.0000000000000316 28002117 PMC5228626

[23] Forsgren MF, Leinhard OD, Dahlström N, Cedersund G, Lundberg P. Physiologically realistic and validated mathematical liver model revels hepatobiliary transfer rates for Gd-EOB-DTPA using human DCE-MRI data. PLoS ONE. 2014;9(4):e104570 10.1371/journal.pone.0095700 24748411 PMC3991717

[24] Karlsson M, Janzén DLI, Durrieu L, Colman-Lerner A, Kjellsson MC, Cedersund G. Nonlinear mixed-effects modelling for single cell estimation: when, why, and how to use it. BMC Syst Biol. 2015;9(1):1–15. 10.1186/s12918-015-0203-x 26335227 PMC4559169

[25] Andersson T, Romu T, Karlsson A, Noren B, Forsgren MF, Smedby O, et al Consistent intensity inhomogeneity correction in water-fat MRI. J Magn Reson Imaging. 2015;42(2):468–76. 10.1002/jmri.24778 25355066

[26] Romu T, Dahlström N, Leinhard OD, Borga M. Robust water fat separated dual-echo MRI by phase-sensitive reconstruction. Magn Reson Med. 2017;78(3):1208–16. 10.1002/mrm.26488 27775180

[27] Shuter B, Tofts PS, Wang SC, Pope JM. The relaxivity of Gd-EOB-DTPA and Gd-DTPA in liver and kidney of the Wistar rat. Magnetic Resonance Imaging. 1996;14(3):243–53. 10.1016/0730-725X(95)02097-D 8725190

[28] Saito K, Ledsam J, Sourbron S, Otaka J, Araki Y, Akata S, et al Assessing liver function using dynamic Gd-EOB-DTPA-enhanced MRI with a standard 5-phase imaging protocol. J Magn Reson Imaging. 2013;37(5):1109–14. 10.1002/jmri.23907 23086736

[29] Juluru K, Talal AH, Yantiss RK, Spincemaille P, Weidman EK, Giambrone AE, et al Diagnostic accuracy of intracellular uptake rates calculated using dynamic Gd‐EOB‐DTPA‐enhanced MRI for hepatic fibrosis stage. J Magn Reson Imaging. 2016;45(4):1177–85. 10.1002/jmri.25431 27527820 PMC5313385

[30] Leporq B, Daire J-L, Pastor CM, Deltenre P, Sempoux C, Schmidt S, et al Quantification of hepatic perfusion and hepatocyte function with dynamic gadoxetic acid-enhanced MR imaging in patients with chronic liver disease. Clin Sci. 2018;132(7):813–24. 10.1042/CS20171131 29440620

[31] Tsuda N, Matsui O. Cirrhotic Rat Liver: Reference to Transporter Activity and Morphologic Changes in Bile Canaliculi—Gadoxetic Acid–enhanced MR Imaging. Radiology. 2010;256(3):767–73. 10.1148/radiol.10092065 20663976

[32] Tsuda N, Matsui O. Signal profile on Gd-EOB-DTPA-enhanced MR imaging in non-alcoholic steatohepatitis and liver cirrhosis induced in rats: correlation with transporter expression. Eur Radiol. 2011;21(12):2542–50. 10.1007/s00330-011-2228-x 21830099

[33] Kim J, Kim T, Hong KS, Moon H, Oh I-K, Mok Lee S, et al Pre-Hepatectomy Assessment of Bile Transporter Expression by Gadoxetic Acid-Enhanced MRI in a Rat Model of Liver Cirrhosis. J Invest Surg. 2016;30(4):265–71. 10.1080/08941939.2016.1238983 27780379

[34] Lagadec M, Doblas S, Giraudeau C, Ronot M, Lambert SA, Fasseu M, et al Advanced fibrosis: correlation between pharmacokinetic parameters at dynamic gadoxetate-enhanced MR imaging and hepatocyte organic anion transporter expression in rat liver. Radiology. 2014;274(2):379–86. 10.1148/radiol.14140313 25289480

[35] Bonin S, Pascolo L, Crocé LS, Stanta G, Tiribelli C. Gene expression of ABC proteins in hepatocellular carcinoma, perineoplastic tissue, and liver diseases. Mol Med. 2002;8(6):318–25. 10.1007/BF03402158 12428063 PMC2039997

[36] Wang L, Collins C, Kelly EJ, Chu X, Ray AS, Salphati L, et al Transporter expression in liver tissue from subjects with alcoholic or hepatitis C cirrhosis quantified by targeted quantitative proteomics. Drug metabolism and disposition: the biological fate of chemicals. 2016;44(11):1752–8. 10.1124/dmd.116.071050 27543206 PMC5074470

[37] Kullak-Ublick GA, Baretton GB, Oswald M, Renner EL, Paumgartner G, Beuers U. Expression of the hepatocyte canalicular multidrug resistance protein (MRP2) in primary biliary cirrhosis. Hepatol Res. 2002;23(1):78–82. 10.1016/S1386-6346(01)00159-0 12084558

[38] Besa C, Lewis S, Pandharipande PV, Chhatwal J, Kamath A, Cooper N, et al Hepatocellular carcinoma detection: diagnostic performance of a simulated abbreviated MRI protocol combining diffusion-weighted and T1-weighted imaging at the delayed phase post gadoxetic acid. Abdom Radiol. 2017;42(1):179–90. 10.1007/s00261-016-0841-5 27448609

[39] Marks RM, Ryan A, Heba ER, Tang A, Wolfson TJ, Gamst AC, et al Diagnostic per-patient accuracy of an abbreviated hepatobiliary phase gadoxetic acid-enhanced MRI for hepatocellular carcinoma surveillance. AJR Am J Roentgenol. 2015;204(3):527–35. 10.2214/AJR.14.12986 25714281

[40] Berndt N, Bulik S, Wallach I, Wunsch T, Konig M, Stockmann M, et al HEPATOKIN1 is a biochemistry-based model of liver metabolism for applications in medicine and pharmacology. Nature communications. 2018;9(1):2386 10.1038/s41467-018-04720-9 29921957 PMC6008457

[41] Nyman E, Rozendaal YJ, Helmlinger G, Hamren B, Kjellsson MC, Stralfors P, et al Requirements for multi-level systems pharmacology models to reach end-usage: the case of type 2 diabetes. Interface focus. 2016;6(2):20150075 10.1098/rsfs.2015.0075 27051506 PMC4759745

[42] Brannmark C, Nyman E, Fagerholm S, Bergenholm L, Ekstrand EM, Cedersund G, et al Insulin signaling in type 2 diabetes: experimental and modeling analyses reveal mechanisms of insulin resistance in human adipocytes. The Journal of biological chemistry. 2013;288(14):9867–80. 10.1074/jbc.M112.432062 23400783 PMC3617287

[43] Maldonado EM, Fisher CP, Mazzatti DJ, Barber AL, Tindall MJ, Plant NJ, et al Multi-scale, whole-system models of liver metabolic adaptation to fat and sugar in non-alcoholic fatty liver disease. NPJ Syst Biol Appl. 2018;4:33 10.1038/s41540-018-0070-3 30131870 PMC6102210

[44] Dixon WT. Simple proton spectroscopic imaging. Radiology. 1984;153(1):189–94. 10.1148/radiology.153.1.6089263 6089263

[45] Pruessmann KP, Weiger M, Scheidegger MB, Boesiger P. SENSE: sensitivity encoding for fast MRI. Magn Reson Med. 1999;42(5):952–62. 10.1002/(SICI)1522-2594(199911)42:5<952::AID-MRM16>3.0.CO;2-S 10542355

[46] Leinhard OD, Dahlström N, Kihlberg J, Sandström P, Brismar T, Smedby Ö, et al Quantifying differences in hepatic uptake of the liver specific contrast agents Gd-EOB-DTPA and Gd-BOPTA: a pilot study. Eur Radiol. 2012;22(3):642–53. 10.1007/s00330-011-2302-4 21984449

[47] Delyon B, Lavielle M, Moulines E. Convergence of a stochastic approximation version of the EM algorithm. Ann Stat. 1999:94–128. 10.1214/aos/1018031103

[48] Batts KP, Ludwig J. An Update on Terminology and Reporting. Am J Surg Pathol. 1995;19(12):1409–17. 7503362 10.1097/00000478-199512000-00007

